# Vasopressor Use in Acute Spinal Cord Injury: Current Evidence and Clinical Implications

**DOI:** 10.3390/jcm14030902

**Published:** 2025-01-29

**Authors:** Mazen Taman, Hael Abdulrazeq, Carlin Chuck, Rahul A. Sastry, Rohaid Ali, Clark C. Chen, Athar N. Malik, Patricia Leigh Zadnik Sullivan, Adetokunbo Oyelese, Ziya L. Gokaslan, Jared S. Fridley

**Affiliations:** Department of Neurosurgery, The Warren Alpert Medical School of Brown University, Providence, RI 02903, USA

**Keywords:** acute spinal cord injury, vasopressors, hemodynamic management, mean arterial pressure (MAP), neurogenic shock, spinal cord perfusion pressure (SCPP), duroplasty

## Abstract

Acute spinal cord injury (SCI) often results in severe neurologic deficits, with hemodynamic instability contributing to secondary ischemic damage. Beyond surgical decompression, maintaining adequate mean arterial pressure (MAP) is key to enhancing spinal cord perfusion and oxygenation. Vasopressor therapy is frequently used to achieve hemodynamic stability, but optimal MAP targets and vasopressor selection remain controversial. This review explores updated guidelines and current evidence regarding MAP management and the use of vasopressors in SCI, focusing on their impact on spinal cord perfusion and neurologic outcomes. Recent studies highlight the role of durotomy in directly improving spinal cord perfusion pressure (SCPP) by reducing intraspinal pressure (ISP), offering a complementary mechanical intervention as part of pharmacologic therapies. Recent guidelines suggest an MAP range of 75–80 mmHg as a lower limit and 90–95 mmHg as an upper limit for 3–7 days post-injury, highlighting the need for personalized hemodynamic management. Norepinephrine is commonly preferred due to its balanced effects on peripheral vascular resistance and spinal cord perfusion pressure (SCPP), though dopamine, phenylephrine, and dobutamine each offer unique hemodynamic profiles suited to specific clinical scenarios. Despite their benefits, vasopressors carry significant risks, including arrhythmias and potential myocardial strain, necessitating careful selection based on individual patient factors. Further research is needed to refine vasopressor use and establish evidence-based protocols that optimize neurologic recovery, alongside continued exploration of SCPP as a potential therapeutic target.

## 1. Introduction

Acute spinal cord injury (SCI) is a serious condition that can result in permanent loss of neurologic function within the first few days following an injury to the spinal cord. Beyond urgent surgical decompression, augmenting mean arterial pressure (MAP) remains one of the few medical treatments available to enhance spinal cord perfusion and oxygen delivery. This approach aims to reduce secondary ischemic damage to vulnerable surviving neural tissue and improve neurologic outcomes [1,2,3,4,5]. Hemodynamic instability, and in particular systemic hypotension, defined as systolic blood pressure below 90 mmHg, is harmful to the injured spinal cord and must be promptly corrected [1]. Ischemia and hypoperfusion play a central role in the progression of secondary injury mechanisms after acute SCI, making the optimization of spinal cord perfusion a key focus in the literature [6,7].

The spinal cord tissue’s vulnerability to ischemia is supported by findings that small changes in MAP and spinal cord perfusion pressure (SCPP), which is the difference between MAP and intrathecal pressure (ITP), are correlated with neurologic outcomes after SCI [8]. Studies have demonstrated that a difference of merely 2–3 mmHg in MAP over the first three days post-injury could distinguish between patients who did not recover neurologically and those who improved by at least one American Spinal Cord Injury Association (ASIA) Impairment Scale (AIS) grade, underscoring the critical therapeutic effects of maintaining MAP thresholds in the early acute period of SCI [5,9]. Furthermore, maintaining adequate MAP has been strongly associated with improvements in the AIS motor subscore, and emerging evidence suggests potential benefits for sensory subscores, highlighting the vital role of hemodynamic optimization in promoting spinal cord perfusion and comprehensive neurological recovery [10,11].

The 2013 guidelines from the American Association of Neurological Surgeons (AANS) and the Congress of Neurological Surgeons (CNS) provided detailed recommendations for the hemodynamic management of acute SCI, emphasizing the maintenance of an appropriate MAP to enhance spinal cord perfusion and improve neurologic outcomes [6]. However, recent advancements and accumulating evidence have prompted a re-evaluation of this approach [1]. This review aims to consider the updated 2024 guidelines, the use of SCPP as a marker for spinal cord perfusion, and the revised understanding of the utilization of vasopressors such as dopamine, norepinephrine, phenylephrine, and dobutamine in the management of acute SCI, emphasizing evolving strategies in hemodynamic optimization [1].

## 2. AANS/CNS Joint Committee Guidelines

The 2013 AANS/CNS joint committee guidelines for the hemodynamic management of acute SCI provided three key level III recommendations: utilizing monitoring devices in a critical care setting to detect cardiovascular and respiratory issues, promptly treating systolic hypotension below 90 mmHg, and maintaining an MAP of 85–90 mmHg for the first seven days post-injury [6]. However, these guidelines required revision due to several limitations, such as a lack of guidance on vasopressor choice, reliance on low-quality evidence, and the emergence of new data [12,13].

The narrow MAP range of 85–90 mmHg proved challenging to maintain with conventional vasopressors, leading many centers to adopt an MAP goal of at least 85 mmHg without an upper limit [14,15]. This prompted the need for clearer, more flexible recommendations that account for clinical variability. While the first two recommendations remain mainstays in acute SCI management, the 2024 guidelines now suggest a broader MAP target range, recommending 75–80 mmHg as the lower limit and 90–95 mmHg as the upper limit for 3–7 days of treatment [1]. The guidelines still offer no specific targets for SCPP or vasopressor choice due to the scarcity of available evidence.

Several studies informed these updates by examining MAP targets in relation to neurologic recovery from SCI. Neurologic recovery was typically measured using the AIS grade or motor score at different intervals, from discharge to one-year post-injury. Consistent findings show that MAPs below 85 mmHg are associated with poorer neurological outcomes in patients with AIS grades A, B, and C [14,16]. Other studies identified varying thresholds between 50 and 80 mmHg, below which neurological deficits worsened, including intraoperatively [9,11,17,18,19,20]. There is conflicting evidence regarding higher MAP targets; while some studies suggest improved outcomes with MAPs above 85 mmHg over three days, others indicate that neurological improvements plateau beyond 95 mmHg, offering limited additional benefit [9,19,21,22,23]. Given the limited support for higher MAP targets, the guideline development group (GDG) recommended not exceeding 90–95 mmHg, citing potential risks from vasopressor use [1]. These flexible target ranges allow clinicians to tailor MAP goals based on individual patient needs and clinical circumstances [1,24].

## 3. Management of Acute Spinal Cord Injury

### 3.1. Hemodynamic Autoregulation

Following acute SCI, the body’s autoregulatory mechanisms for maintaining adequate blood flow to the spinal cord are often severely compromised, leading to dysregulated blood pressure and impaired spinal cord perfusion [2,25]. Normally, the spinal cord’s blood supply is tightly regulated by autoregulatory processes that ensure stable perfusion despite fluctuations in systemic blood pressure [26]. This regulation involves a complex interplay of neural, humoral, and local factors that adjust vascular tone in response to changes in blood flow and pressure [25,26]. However, after acute SCI, these mechanisms are disrupted due to direct damage to the spinal cord, leading to systemic hypotension, bradycardia, and vasodilation below the level of injury, which together impair the spinal cord’s ability to autoregulate its blood supply [25,26,27].

The pathophysiology of dysregulated blood pressure in SCI is multifactorial. The loss of sympathetic tone due to injury disrupts the normal balance between vasoconstriction and vasodilation, leading to unopposed parasympathetic activity [26]. This results in profound vasodilation, particularly in the vascular beds below the level of injury, contributing to hypotension. Additionally, the spinal cord itself becomes more vulnerable to ischemia as autoregulatory responses to maintain perfusion pressure are lost [2]. The compromised blood–brain barrier and local edema further exacerbate this situation, leading to secondary ischemic injury. The failure to maintain an adequate MAP and SCPP can therefore lead to irreversible damage to neural tissue and poor neurological outcomes [2,26].

### 3.2. Regional Vulnerability to Ischemia

The vulnerability of different regions of the spinal cord to ischemia is influenced by both anatomical and pathophysiological factors, highlighting the need for nuanced hemodynamic management based on injury characteristics (Table 1) [28,29]. The spinal cord’s vascularization is primarily supplied by the anterior spinal artery (0.2–0.8 mm), the posterolateral spinal artery (0.1–0.4 mm), and the arteria radicularis magna or the artery of Adamkiewicz (0.5–1.2 mm) [28,29,30,31]. The cervical spinal cord, with its relatively greater metabolic demand and reliance on the anterior spinal artery, may be more susceptible to ischemia compared to the thoracic or lumbar segments [4,30,31,32]. The elevated metabolic activity of the cervical spinal cord is attributed to the high density of motor neurons within the cervical enlargement, which facilitates the complex innervation of the upper limbs [33,34]. Additionally, the cervical cord handles a substantial volume of ascending sensory and descending motor pathways due to its anatomical proximity to the brain, further intensifying its metabolic requirements. Variations in vascular supply also contribute to differing injury presentations, such as anterior spinal artery syndrome, which results in motor deficits and a loss of pain and temperature sensation, or posterior spinal artery syndrome, which affects proprioception and vibratory sense [30,31,32]. Less common presentations, such as sulcal artery syndrome, conus medullaris infarction (potentially misdiagnosed as cauda equina syndrome), and central spinal infarction (often post-cardiac arrest or severe prolonged hypotension), further illustrate the complexity of ischemic SCI [4,28,30].

The mechanism of SCI also significantly influences the pattern and severity of ischemic injury. Traumatic SCIs frequently involve direct vascular disruption, mechanical compression, and immediate ischemia, whereas non-traumatic SCIs, such as those due to vascular insufficiency or degenerative conditions, may cause more gradual ischemic insults [4,29,37,38,39]. These differences underscore the necessity of tailoring hemodynamic interventions, such as MAP targets and vasopressor selection, based on the mechanism and neurological level of injury. Current evidence predominantly addresses traumatic cervical SCIs, which often result in neurogenic shock characterized by profound bradycardia, hypotension, and autonomic dysregulation [4,29,30,31]. In contrast, thoracic or lumbar SCIs, while less likely to produce neurogenic shock, may necessitate distinct strategies, such as preventing venous stasis and maintaining systemic perfusion [4,29,30,31].

Cervical and high thoracic injuries disrupt sympathetic outflow while preserving parasympathetic activity, resulting in bradycardia, reduced cardiac output, and hypotension, especially during exertion or upright positioning [4,30]. A loss of vascular tone causes peripheral blood pooling and reduced venous return. These patients are at a high risk of autonomic dysreflexia, a life-threatening syndrome triggered by noxious stimuli, characterized by sudden hypertension below the injury and compensatory vasodilation above [4,30]. Effective management requires vigilant monitoring, vasopressor use to maintain MAP, and the prevention of triggers. The heterogeneity in presentations and complications between cervical, thoracic, and lumbar injuries reflects the complexity of SCI pathophysiology, where both primary and secondary injury mechanisms—ischemia, inflammation, and glial scar formation—differ across injury types and levels [4,29,30,37].

### 3.3. Vasopressor and Inotrope Selection

In managing acute SCI, vasopressors and inotropes are employed to counteract hemodynamic disturbances by increasing systemic blood pressure and improving spinal cord perfusion [2]. This complex process requires a nuanced understanding of the pathophysiology of neurogenic shock and the pharmacologic profiles of available agents, such as norepinephrine, dopamine, phenylephrine, and dobutamine. Vasopressors, including norepinephrine and phenylephrine, primarily increase systemic vascular resistance through alpha-adrenergic vasoconstriction, supporting blood pressure and perfusion. In contrast, inotropes like dobutamine enhance myocardial contractility via beta-1 adrenergic stimulation, improving cardiac output. Some agents, like dopamine and epinephrine, exhibit mixed vasopressor and inotropic effects based on dose and receptor activity. While vasopressors are typically used to maintain MAP, inotropes are reserved for patients with cardiac dysfunction. Tailored use of these agents is essential for addressing the unique hemodynamic challenges in SCI (Table 2).

Norepinephrine is frequently the first-line vasopressor in acute SCI management due to its potent alpha-adrenergic agonist effects, which increase peripheral vascular resistance and counteract the severe hypotension characteristic of neurogenic shock [18,26,40,41]. Its mild beta-adrenergic activity also slightly enhances heart rate and myocardial contractility, making it a versatile choice, especially in patients who might benefit from a modest boost in cardiac output [40,41]. Notably, norepinephrine’s ability to maintain or even reduce intrathecal pressure supports SCPP more effectively than other vasopressors, providing a distinct advantage in SCI management [42,43,44]. For example, when compared with dopamine, norepinephrine provided a 2 mmHg increase in spinal cord perfusion pressure without differential MAP effects [42]. However, its potent vasoconstrictive effects necessitate cautious use in patients with peripheral vascular disease due to the potential exacerbation of ischemic conditions [40,41,42,44]. Additionally, prolonged use requires central venous access due to the risk of local tissue necrosis if administered peripherally [41,43,48]. In animal models, norepinephrine has also demonstrated superiority over phenylephrine in restoring spinal cord blood flow and oxygenation, though translating these findings to human clinical practice remains complex [44].

Dopamine is another commonly used vasopressor, with dose-dependent effects on dopaminergic, beta-adrenergic, and alpha-adrenergic receptors. At low doses, dopamine’s stimulation of dopaminergic receptors causes renal and mesenteric vasodilation, which may be counterproductive in the context of SCI due to potential reductions in systemic blood pressure [45]. Moderate doses activate beta-adrenergic receptors, enhancing heart rate and myocardial contractility, which is beneficial in patients with concurrent heart dysfunction [45]. At higher doses, dopamine predominantly exerts alpha-adrenergic effects, increasing vascular resistance [45]. However, dopamine is associated with increased intrathecal pressure and reduced SCPP, potentially worsening neurological outcomes, and its use has been linked to higher rates of arrhythmias, particularly in older patients [1,40,42,45,49,50]. Due to its higher complication rates compared to other vasopressors, including an elevated risk of myocardial injury and arrhythmias, dopamine is generally less favored, especially in elderly patients. Prolonged administration also requires central access, similar to norepinephrine. Dopamine’s contraindication in patients with pheochromocytoma further limits its utility.

Epinephrine, a potent beta and alpha agonist, significantly increases heart rate, cardiac output, and systemic vascular resistance, providing broad-spectrum cardiovascular support [18,41]. However, these effects also come with a higher risk of complications, such as tachyarrhythmias and increased myocardial oxygen demand, which can be particularly challenging in SCI patients [18,26]. Due to its extensive side-effect profile and pronounced impact on myocardial workload, epinephrine is generally reserved for cases of persistent refractory hypotension when other vasopressors fail to achieve adequate hemodynamic stability [18,26]. Its use is limited to situations where its potent effects are necessary, but careful monitoring is essential to mitigate the associated risks.

Phenylephrine, a selective alpha-1 adrenergic agonist, acts as a potent vasoconstrictor and is particularly effective when an increase in systemic vascular resistance is needed without affecting heart rate [26,46]. However, phenylephrine’s lack of beta-adrenergic activity means it does not enhance cardiac output, which can be a limitation in patients with impaired myocardial function. Phenylephrine is often reserved for situations where other vasopressors are contraindicated or in patients with tachyarrhythmias that limit the use of norepinephrine [43,44]. Central venous access is required for prolonged use due to the risk of extravasation and tissue damage. Although phenylephrine is generally well tolerated, it should be used cautiously in patients with severe bradycardia or heart block, as its effects can exacerbate these conditions [41,44,46]. Experimental data suggest that norepinephrine may be more effective than phenylephrine in improving spinal cord perfusion and oxygenation, further supporting the tailored use of vasopressors based on patient-specific needs.

Dobutamine primarily stimulates beta-1 adrenergic receptors, enhancing myocardial contractility and cardiac output, making it particularly useful in patients with concurrent heart dysfunction [51,52]. Its weak alpha-1 adrenergic activity induces limited vasoconstriction, while beta-2 receptor stimulation offers mild vasodilation [51]. This vasodilatory effect, coupled with an elevated risk of tachyarrhythmias and increased myocardial oxygen demand, necessitates cautious use in patients with significant cardiac risks [48,53]. Unlike norepinephrine and phenylephrine, dobutamine does not significantly increase systemic vascular resistance, which limits its use as a standalone agent for managing hypotension due to neurogenic shock. However, it is often combined with other vasopressors to balance its effects, particularly in patients who need improved cardiac output. Dobutamine generally does not require central venous access unless used at high doses or for extended periods, reducing some logistical challenges compared to other agents [51,52]. It is preferred in patients with heart failure or significant myocardial dysfunction and may be a better choice than norepinephrine for restoring cardiac output and spinal cord oxygenation in these patients [48].

### 3.4. Additional Considerations

While essential for maintaining MAP in the management of acute SCI, vasopressor therapy is associated with significant risks. Studies have reported high rates of complications when using vasopressors to sustain MAP above 85 mmHg in critically ill patients, with 74–94% of patients experiencing tachycardia, bradycardia, atrial fibrillation, ventricular tachycardia, and elevated troponin levels [14,40,49,50]. Although most of these complications were not life-threatening, vasopressors can increase the risk of severe outcomes, such as fatal cardiac arrhythmias, myocardial injury, acidosis, and skin necrosis [49,50].

Additionally, vasopressor therapy has been linked to complications beyond direct cardiovascular effects. Evidence suggests that the use of vasopressors is independently associated with higher rates of infections and procedural complications, including pneumonia, deep vein thrombosis, unplanned surgical interventions, and surgical site infections, indicating a broader pharmacologic impact beyond blood pressure control [23]. However, some studies have found that maintaining MAP above 85 mmHg for shorter durations of 3–7 days does not significantly increase the risk of severe events such as hemorrhage, stroke, or myocardial infarction, highlighting the importance of carefully balancing the duration and intensity of vasopressor use to minimize potential harm [13,23].

## 4. Spinal Cord Perfusion Pressure

While MAP targets have been extensively studied in the management of acute SCI, emerging evidence suggests that SCPP may be a more critical determinant of neurologic recovery [54,55]. Increased SCPP has been linked to significant improvements in neurologic outcomes at 6 and 12 months, underscoring its potential role in enhancing neural tissue perfusion and oxygenation after SCI [16,19,54,56,57,58]. Although MAP augmentation aims to improve spinal cord perfusion indirectly, directly targeting SCPP may offer a more precise approach to mitigating secondary ischemic injury.

Duroplasty has emerged as a pivotal intervention to augment SCPP in patients with acute SCI by effectively reducing intraspinal pressure (ISP) [59,60]. Traditional methods, such as laminectomy alone, have been found inadequate for relieving ISP or sufficiently increasing SCPP, as the dura itself frequently serves as a source of compression on the injured spinal cord [59]. In contrast, duroplasty, which involves dural decompression and the application of an artificial dural patch, has demonstrated significantly improved critical physiological parameters, including ISP and SCPP [59,60].

Moreover, combining laminectomy with duroplasty has been shown to significantly expand the intradural space, leading to more effective and sustained decompression of the spinal cord when compared to laminectomy alone [59]. This is evidenced by marked improvements in SCPP and reductions in ISP and the vascular pressure reactivity index (sPRx), indicating improved spinal cord vascular responsiveness which may aid in preventing secondary ischemic injury [59]. Furthermore, ongoing phase III randomized controlled trials, such as the Duroplasty for Injured Cervical Spinal Cord with Uncontrolled Swelling (DISCUS) trial, reinforce the potential of duroplasty to not only improve early post-injury SCPP but also promote long-term functional recovery, solidifying its role as a critical component in the surgical management of acute SCI [61].

However, SCPP monitoring presents challenges due to its invasive nature, requiring intradural catheters that can result in complications such as cerebrospinal fluid (CSF) leakage and pseudomeningocele [19,21,43,54,62,63]. Despite these risks, studies have reported minimal significant complications associated with SCPP monitoring and CSF drainage, suggesting it remains a viable adjunct in select cases. Research by Papadopoulos and colleagues involving pressure sensors placed at the injury site to detect spinal cord swelling revealed that the “optimal SCPP” varies among patients, further emphasizing the need for individualized approaches [19,58]. However, direct clinical application remains limited, and standardization of these techniques is not yet widespread.

Though promising, the optimal SCPP target range is still under investigation, with studies suggesting that maintaining SCPP above 50 mmHg but below 110 mmHg may be beneficial [19,54,64,65]. Comparisons between SCPP and MAP indicate that SCPP correlates more closely with improved neurologic recovery, whereas relying solely on MAP targets may not be as effective [54,58,64]. Given the lack of established SCPP targets, practical difficulties in measurement, and variable techniques, current guidelines continue to recommend focusing on MAP targets as a more accessible and widely accepted approach for managing acute SCI [1,2,54,66]. This pragmatic stance reflects the ongoing need for further research to refine SCPP monitoring techniques and mechanical interventions like duroplasty, alongside vasopressor use, to better define their roles in optimizing outcomes for SCI patients.

## 5. Discussion

The updated CNS/AANS guidelines on the hemodynamic management of acute SCI provide weak recommendations for maintaining MAP between 75–80 mmHg as a lower limit and 90–95 mmHg as an upper limit, with a treatment duration of 3–7 days [1]. These recommendations reflect the low quality of evidence currently available, as well as the recognized impact that small fluctuations in MAP and SCPP can have on neurologic outcomes due to the spinal cord’s heightened vulnerability to ischemia. Despite these guidelines, significant challenges remain in establishing precise MAP targets that consistently optimize neurologic recovery across diverse patient populations.

Importantly, current guidelines are predominantly derived from studies focusing on traumatic cervical SCIs, leaving significant gaps in our understanding of optimal hemodynamic management for other injury mechanisms and levels, such as non-traumatic SCIs or injuries to the thoracic and lumbar spine. These variations in vascular supply, metabolic demand, and injury presentations highlight the need for broader evidence to develop comprehensive recommendations addressing the heterogeneity of SCI pathophysiology.

The existing literature on MAP targets in SCI management is limited by several key factors. Most studies report associations rather than causative relationships between MAP thresholds and recovery outcomes, making it difficult to draw definitive conclusions. Additionally, patient-specific factors such as AIS grade, the presence of concomitant injuries, and underlying comorbidities can significantly alter the impact of MAP on neurologic outcomes, further complicating the interpretation of data. Many of the studies reviewed had small sample sizes, restricted patient populations, and often focused on specific post-injury timeframes, limiting the generalizability of their findings to the broader SCI patient population. The previous 2013 guidelines suggested maintaining MAP between 85 and 90 mmHg, but the inconsistency and variability in the lower-limit recommendations in the studies they were based on underscore the need for more robust evidence to refine these targets.

Regarding the optimal duration of hemodynamic management, evidence suggests that supporting MAP during the early days post-injury is critical, with some studies indicating that extending this support up to 7 days can further enhance neurologic outcomes [9,11,12,14,18,20,22,49,50]. However, these studies often do not fully address the impact of varying injury severities and individual hemodynamic requirements, highlighting the need for a flexible approach. As a result, the GDG recommended a treatment duration range of 3–7 days, allowing clinicians to tailor the duration based on patient-specific needs, injury characteristics, and clinical response [1]. In addition to pharmacological interventions like vasopressors, mechanical interventions such as duroplasty have shown promise in directly improving SCPP by reducing intraspinal pressure and relieving compression, further optimizing perfusion and contributing to better neurologic outcomes.

The choice of vasopressor or inotrope in SCI management must be tailored to the patient’s specific clinical condition, considering factors such as the level and severity of injury, the presence of cardiopulmonary dysfunction, and comorbidities. For example, patients with pre-existing heart conditions may benefit more from agents like dobutamine, which improve cardiac output, while those with significant hypotension may require the potent vasoconstrictive effects of norepinephrine or phenylephrine. Additionally, the risk of complications such as arrhythmias, myocardial injury, and tissue necrosis must be weighed against the benefits of MAP augmentation. Central venous access is often required for the prolonged administration of these agents to minimize the risk of extravasation and local tissue damage, particularly with drugs like norepinephrine and phenylephrine. Ultimately, a personalized approach that considers patient-specific factors, such as the presence of a central line, concurrent cardiopulmonary dysfunction, and comorbidities, along with close monitoring for complications, is warranted when using these agents to manage acute SCI [1].

A major knowledge gap persists regarding the optimal choice of vasopressor for managing hemodynamics in SCI patients. Current evidence does not strongly favor one vasopressor over another, leaving clinicians to make decisions based on personal experience, institutional protocols, and patient-specific considerations. Addressing this gap through targeted research is essential for developing evidence-based guidelines that will refine vasopressor selection, improve hemodynamic management, and ultimately enhance neurologic outcomes in SCI patients. Expanding our understanding of how different vasopressors affect spinal cord perfusion and recovery is crucial to advancing the care of patients with acute SCI.

## 6. Conclusions

Acute SCI requires precise hemodynamic management to optimize spinal cord perfusion and prevent secondary ischemic injury. The updated CNS/AANS guidelines recommend maintaining MAP between 75–80 mmHg as a lower limit and 90–95 mmHg as an upper limit for 3–7 days, reflecting the limitations of current evidence and the need for individualized care based on patient-specific responses. While MAP remains a central focus of treatment, emerging evidence suggests that directly targeting SCPP, particularly through interventions like duroplasty, could further enhance outcomes by alleviating intraspinal pressure and improving perfusion.

Vasopressor selection remains a crucial yet complex aspect of managing acute SCI, as no single agent has been definitively proven superior in improving outcomes. Norepinephrine is frequently favored for its balanced effects on both MAP and SCPP, but the choice of agent must be tailored to individual patient profiles, considering factors such as cardiac function, injury severity, and comorbidities. Other agents, such as dopamine, phenylephrine, and dobutamine, may offer distinct advantages depending on the clinical scenario, reinforcing the need for a personalized approach to hemodynamic management.

Ongoing research is critical to advancing our understanding of both pharmacologic and mechanical strategies in acute SCI care. As evidence evolves, refining vasopressor protocols and integrating interventions like duroplasty will be essential for establishing comprehensive, evidence-based guidelines that maximize neurologic recovery and long-term outcomes for SCI patients.

## Figures and Tables

**Table 1:** Arterial and venous supply of the cervical and thoracic spinal cord (C1–T12) [35,36].

Region	Vessel	Vascular Anatomy	Clinical Relevance
Cervical (C1–C8)	Anterior Spinal Artery (ASA)	Arises from the vertebral arteries, supplying the two anterior thirds of the spinal cord.	Cervical segments are vulnerable to ischemia during hypotension or arterial compression. Posterior cervical spinal procedures risk injury to vertebral arteries. Impaired drainage through the IVVP can elevate intraspinal pressure, contributing to cord edema and worsening secondary injury.
	Posterior Spinal Arteries (PSAs)	Paired arteries originating from the vertebral or posterior inferior cerebellar arteries (PICAs), supplying the posterior third of the spinal cord.	
	Radicular Arteries	Segmental contributions from the vertebral and ascending cervical arteries.	
	Anterior Spinal Vein	Drains the two anterior thirds of the cord.	
	Posterior Spinal Veins	Paired veins draining the posterior third of the spinal cord.	
	Radicular Veins	Drain into the internal vertebral venous plexus (IVVP).	
	IVVP	Connects to external venous systems, including the vertebral and jugular veins.	
Upper Thoracic (T1–T6)	ASA and PSAs	Continue to supply anterior and posterior regions, respectively.	The limited collateral arterial supply increases the risk of ischemic injury during systemic hypoperfusion. Thoracic surgeries, particularly spine or aortic procedures, may damage radiculomedullary arteries and can lead to anterior spinal artery syndrome. Congestion in the azygos system may exacerbate cord edema or secondary injury.
	Radiculomedullary Arteries	Arise from the intercostal arteries, providing collateral support.	
	Segmental Arteries	Arise from the thoracic aorta, feeding radicular branches.	
	Anterior and Posterior Spinal Veins	Continue their roles from the cervical region.	
	Radicular Veins	Drain into the azygos and hemiazygos systems.	
	IVVP	Communicates with systemic venous systems, aiding in drainage.	
Lower Thoracic (T7–T12)	ASA	Narrower in diameter, requiring reinforcement from the artery of Adamkiewicz (AKA)	Injury to the AKA, particularly during thoracoabdominal aortic surgery, can result in anterior spinal artery syndrome with paralysis and motor deficits. This leaves the thoracolumbar region highly susceptible to ischemic injury due to its reliance on a single dominant arterial supply. Venous drainage challenges: compression or disruption in the venous system may lead to impaired perfusion and worsening of spinal cord injury.
	AKA	Arises from the descending aorta (most often on the left at T8–L2).	
	PSAs	Continue as paired longitudinal arteries.	
	Segmental Arteries	Arise from the posterior intercostal arteries and lumbar arteries, providing radicular branches to support spinal cord perfusion.	
	Anterior and Posterior Spinal Veins	Continue to drain their respective regions.	
	Radicular Veins	Connect to the intercostal and lumbar venous systems.	
	IVVP	Extends into the lower thoracic and lumbar regions, with connections to the iliac venous system.	

**Table 2:** Vasopressors used in acute spinal cord injury management [18,40,41,42,43,44,45,46,47].

Vasopressor	Norepinephrine	Epinephrine	Dopamine	Phenylephrine	Dobutamine
Receptor Activity	Alpha-1, Mild Beta-1	Alpha-1, Beta-1, Beta-2	Low dose: Dopaminergic; Moderate dose: Beta-1; High dose: Alpha-1	Selective Alpha-1	Beta-1, Mild Beta-2, Mild Alpha-1
Physiological Effects	Increases PVR, slight increase in HR and CO	Increases HR, CO, and PVR	Low dose: Vasodilation; Moderate dose: Increased HR and contractility; High dose: Increased PVR	Increases PVR with minimal effect on HR	Increases PVR, slight increase in HR and CO
Adverse Effects	Arrhythmias, tissue necrosis with extravasation, HTN	Arrhythmias, increased myocardial oxygen demand, HTN	Arrhythmias, increased intrathecal pressure, risk of reduced SCPP, tissue necrosis	Bradycardia, HTN, tissue necrosis with extravasation, reduced CO in heart dysfunction	Arrhythmias, increased myocardial oxygen demand, tissue necrosis with extravasation, HTN
Central Access Required for Prolonged Use	Yes	Yes	Yes	Yes	Typically not required unless at high doses or prolonged use
Precautions	Peripheral vascular disease	Caution in patients with cardiac conditions or arrhythmia risk	Older adults due to arrhythmia risk; Contraindicated in pheochromocytoma	Severe bradycardia or heart block	Severe aortic stenosis, significant tachyarrhythmia
Drug Interactions	Beta-blockers, MAOI	Significant interactions with MAOIs, tricyclic antidepressants	Significant interactions with MAOI, tricyclic antidepressants	Beta-blockers, other vasoconstrictors	Beta-blockers, MAOI
Patient-Specific Considerations	Preferred in patients with shock requiring vasoconstriction	Useful in patients requiring both inotropic and vasoconstrictive effects	Caution in elderly; Avoid in pheochromocytoma	Useful in tachyarrhythmias where HR control is needed	Preferred in patients with heart failure or significant myocardial dysfunction; Use cautiously in patients at risk of tachyarrhythmias or with myocardial ischemia

Abbreviations: PVR: peripheral vascular resistance; HR: heart rate; CO: cardiac output; HTN: hypertension; MAOI: monoamine oxidase inhibitor.

## Data Availability

The original contributions presented in this study are included in the article. Further inquiries can be directed to the corresponding author.
